# HIF-1, the Warburg Effect, and Macrophage/Microglia Polarization Potential Role in COVID-19 Pathogenesis

**DOI:** 10.1155/2021/8841911

**Published:** 2021-03-12

**Authors:** Elisabetta Ferraro, Maria Germanò, Rocco Mollace, Vincenzo Mollace, Natalia Malara

**Affiliations:** ^1^Università di Pisa, Pisa, Italy; ^2^Ospedale Sant'Anna, Como, Italy; ^3^Università Magna Grecia, Catanzaro, Italy

## Abstract

Despite the international scientific community's commitment to improve clinical knowledge about coronavirus disease 2019 (COVID-19), knowledge regarding molecular details remains limited. In this review, we discuss hypoxia's potential role in the pathogenesis of the maladaptive immune reaction against severe acute respiratory syndrome coronavirus-2 (SARS-CoV-2). The state of infection, with serious respiratory dysfunction, causes tissues to become hypoxic due to a discrepancy between cellular O_2_ uptake and consumption similar to that seen within tumor tissue during the progression of numerous solid cancers. In this context, the heterogeneous clinical behavior and the multiorgan deterioration of COVID-19 are discussed as a function of the upregulated expression of the hypoxia-inducible factor-1 (HIF-1) and of the metabolic reprogramming associated with HIF-1 and with a proinflammatory innate immune response activation, independent of the increase in the viral load of SARS-CoV-2. Possible pharmacological strategies targeting O_2_ aimed to improve prognosis are suggested.

## 1. Introduction

Coronavirus disease 2019 (COVID-19) has spread worldwide, causing overwhelming repercussions on daily and working life [1, 2]. The clinical severity of the severe acute respiratory syndrome coronavirus 2 (SARS-CoV-2) infection is determined by the multifaceted biological host response to the harmful virus that could be summarized as the succession of three prevalent pathogenic steps: (i) the direct damage of infected host cells, (ii) the early immune response characterized prevalently by cytokine release and complement activation, and (iii) the persistent inflammation in multiple tissue sites with progressive multiorgan deterioration [3–5]. An additional associated feature of COVID-19 is tissue hypoxia along with overexpression of the hypoxia-inducible factor-1 (HIF-1) along with their immunometabolic and immune-response consequences, which are the focus of this review.

## 2. HIF-1

**Figure 1:**
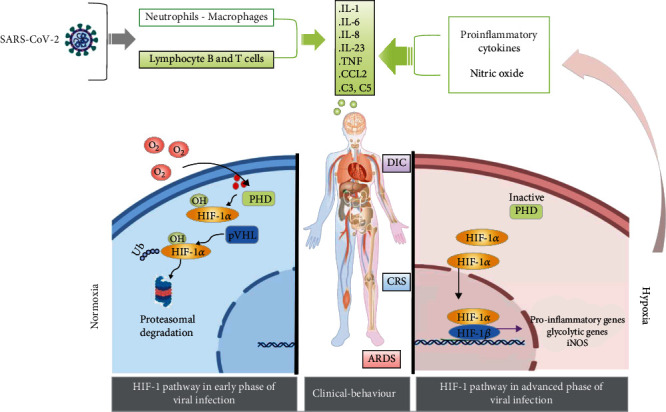
Severe SARS-CoV-2 syndrome as a result of hypoxia and HIF-1 signaling pathway activation. ARDS: acute respiratory distress syndrome; CCL: chemokine (C-C motif) ligand; CRS: cytokine release syndrome; DIC: disseminated intravascular coagulation; HIF-1: hypoxia-inducible factor; IL: interleukin; iNOS: inducible NO synthase; pVHL: von Hippel-Lindau tumor suppressor; SARS-CoV-2: severe acute respiratory syndrome coronavirus; TNF: tumor necrosis factor.

HIFs are a family of highly conserved transcription factors activated by low O_2_ partial pressure (pO_2_). The HIF-1 complex is a heterodimer composed of two basic helix-loop-helix subunits: the HIF-1*α* and the HIF-1*β* subunits. HIF-1*β* is constitutively expressed whereas HIF-1*α* activity is posttranscriptionally regulated by O_2_ sensors and O_2_-dependent enzymes including prolyl hydroxylases (PHDs) [6, 7]. Under normoxia, when O_2_ is available, O_2_-dependent PHDs are active and lead to HIF-1*α* hydroxylation at conserved proline residues. This allows the recognition by the von Hippel-Lindau (pVHL) E3 ubiquitin ligase complex which causes HIF-1*α*'s fast ubiquitination and its proteasomal degradation (Figure 1). Conversely, under hypoxia, PHDs are inhibited since PHDs need O_2_ as a cosubstrate, and this reduces the PHD-dependent degradation of HIF-1*α* resulting in its stabilization; once stabilized, HIF-1*α* dimerizes with HIF-1*β* and binds the promoters at hypoxia-responsive elements, thus inducing the expression of its target genes which are useful in hypoxia conditions. In fact, these target genes are involved not only in O_2_-independent energy production (e.g., glycolytic genes) but also in angiogenesis (e.g., VEGF) and erythropoiesis, thereby increasing O_2_ delivery to tissues [8, 9].

## 3. The Warburg Effect

All cells need a source of energy to maintain homeostasis. Glycolysis and mitochondrial respiration/oxidative phosphorylation (OxPhos) generate energy in the form of ATP. Under normoxic conditions, most cells use the pyruvate obtained by metabolizing glucose through cytosolic glycolysis to feed the Krebs cycle/tricarboxylic acid cycle (TCA). For this purpose, pyruvate enters the mitochondria where it is converted to acetyl coenzyme A (CoA) by the pyruvate dehydrogenase (PDH). Through the TCA cycle, acetyl CoA is oxidized to CO_2_, and reducing equivalents (NADH and FADH_2_) are produced. NADH and FADH_2_ are then used and oxidized by the respiratory chain complexes and O_2_ to generate the mitochondrial membrane proton gradient used by the ATPase to phosphorylate ADP to ATP. The OxPhos leads to the production of 36 molecules of ATP per molecule of glucose. In conditions of O_2_ deficiency (anaerobiosis), the activity of the mitochondrial OxPhos is reduced and the pyruvate deriving from glycolysis is directly converted in the cytosol into lactate; this glycolytic cycle is rapid and allows the formation of few ATP molecules per single molecule of glucose.

Some cells display an enhanced conversion of glucose to pyruvate, this then being metabolized to lactate even in the presence of abundant O_2_; this aerobic glycolysis is called the Warburg effect [10]. In particular, this effect identifies the metabolic state of tumor cells and is highly important in antitumor treatments [11]. However, aerobic glycolysis is a metabolic condition also associated with pluripotency or observed in other cells; some of these belonging to the immune system [12, 13]. The Warburg effect reprogramming includes the overexpression of glucose transporters and glycolytic enzymes as well as the accumulation of glycolytic intermediates and lactate along with environmental acidification. This metabolic condition is characterized by a high-speed ATP production through the fast glycolytic flux and meets the energy demand of rapidly proliferating cells. In fact, the Warburg effect is a common feature of cells which exhibit a rapid proliferation rate and therefore switch to aerobic glycolysis to allow a rapid, often transitory, ATP production. *Vice versa*, the mitochondrial respiration prevalently supports stable and long-lasting processes, e.g., those related to cellular differentiation.

## 4. The Warburg Effect and HIF-1*α* in the Innate Immune Response

The innate immune response is characterized by macrophage (M*Φ*)/microglia cell (MG) activation toward a proinflammatory state which is often identified as M1-like activation. This first proinflammatory step is followed by a step involving anti-inflammatory and proregenerative M*Φ*/MG (M2-like polarized cells). One needs to take into account that this is an oversimplification; indeed, the proinflammatory M1 and the anti-inflammatory M2 M*Φ*/MG populations are the extremes of a wide range of intermediate activation states still not clearly defined.

M1 and M2 subsets express a different sensome and a different secretome; notably, different metabolic pathways are also enrolled by M*Φ*/MG in these two different activation states. In particular, the Warburg effect is typical of rapidly dividing proinflammatory M1 M*Φ*/MG [9]; ATP generation by enhanced aerobic glycolysis is associated with M1's rapid proliferation rate, with increased production of defense factors, with enhanced phagocytosis, and with the antigen-presenting function. Indeed, in these M1 proinflammatory cells as well as in cancer cells, the intracellular transport of glucose increases thanks to an increased expression of the glucose transporter GLUT1. In addition, the enzyme hexokinase, which catalyzes the first step of glycolysis and of the pentose phosphate pathway (PPP), is upregulated under this activation state. M1 subsets rearrange their metabolic flow and modify their intracellular production of ATP relying on glycolysis, necessary for their rapid activation, whereas the OxPhos decreases. The reduced activity of the respiratory chain allows the M1 subsets to employ the O_2_ to produce reactive O_2_ species (ROS) and nitric oxide (NO), whose generation also needs an upregulation of the PPP for the production of NADPH. NADPH is a substrate both for the NADPH oxidases (NOX), which produce ROS, and for inducible NO synthase (iNOS), which produces NO. In fact, another metabolic feature of M1 subsets is the enhanced PPP rate [14]. Moreover, some glycolytic enzymes such as 6-phosphofructo-2-kinase/fructose-2,6-biphosphatase 3 (PFKFB3), pyruvate kinase M2 (PKM2), and *α*-enolase have been found to be crucial in supporting the proinflammatory function. Therefore, glycolysis is also necessary for M1 activation by providing signaling mediators which drive M1 polarization (reviewed by De Santa and collaborators [9]). In addition, although data are not clear and need further elucidation, M1 polarization also features flux discontinuities at several levels of the Krebs cycle, leading to the accumulation or reduction of some TCA intermediates which influence the inflammatory response. In particular, M1 subsets are characterized by an increase of proinflammatory succinate and citrate, immunoresponsive gene 1 (Irg1), isocitrate, and microbicidal itaconic acid and by the downregulation of isocitrate dehydrogenase 1 (Idh1) [9, 15]. Specifically, citrate, succinate, and itaconate are not only consequences but also a cause of M1 polarization.

*Vice versa*, rapid and acute activation is less important for the anti-inflammatory and regenerative M2 M*Φ*/MG whose role lasts longer to ensure proper tissue repair, producing ATP mainly by OxPhos. These observations led to the hypothesis that the OxPhos metabolic phenotype is more suitable for cells involved in long-term reparative roles (anti-inflammatory M2), while the aerobic glycolytic phenotype is necessary to produce rapid and transient responses (proinflammatory M1) [16, 17]. M2 are therefore characterized by a high and efficient OxPhos, which is required for M2 polarization, and also by an intact TCA cycle. The role of fatty acid *β*-oxidation in M2 activation is a matter of debate; in fact, it has been proposed that the overall oxidative metabolism, also fueled by glycolysis and not specifically by fatty acid *β*-oxidation, is crucial for M2 polarization (reviewed by De Santa and coworkers [9]). Finally, glutamine is also vital for M2 activation; the PPP rate decreases in M2 subsets whereas carbohydrate kinase-like (CARKL), a repressor of M1 activation, is upregulated.

During M*Φ*/MG activation, the metabolic adaptation is a key component required for polarization and not only its consequence [18]. It is now widely accepted that the metabolic phenotypic distinction between the M1 and M2 populations also drives the functional diversity of these two cellular effectors of the innate immunity. Paralleling the murine studies, the proinflammatory phenotype of human M1 M*Φ*, leading to an increased production of cytokines such as IL12p40, TNF*α*, or IL-6, is also characterized by an enhanced glycolytic energy pathway [16].

Notably, HIF-1*α* becomes activated during M1 polarization. This also occurs in an O_2_-independent manner, in the presence of O_2_ (normoxia). Indeed, PHD is downregulated in M1 by proinflammatory cytokines and by nuclear factor kB (NF-*κ*B) binding to its promoter. In addition, succinate, which is highly expressed in M1 subsets, inhibits PHD activation, thus stabilizing HIF-1*α* in the presence of O_2_ (the so-called pseudohypoxia). Finally, ROS and NO reduce PHD activity and also promote HIF-1*α* expression under normoxia [9].

HIF-1 plays a crucial role in orchestrating part of the M1 polarization, since it enhances the expression of the proinflammatory IL-1*β* and of other proinflammatory genes [9], downregulates the M2 marker CD206, and also induces iNOS expression. In addition, HIF-1 typically stimulates glucose uptake and the expression of key glycolytic enzymes as well as of pyruvate dehydrogenase kinase-1 (PDK1), thus promoting the metabolic reprogramming leading to M1 polarization [19–21]. In M1 subsets, besides being normoxic, the activation of HIF-1 transcription might also be typically hypoxic; in fact, M1 M*Φ*/MG are likely to be exposed to hypoxic environments during an infection. In addition, HIF-1 promotes stemness and stem cells reside within hypoxic regions. HIF-1 is involved in their homeostasis by decreasing their reliance on oxidative metabolism; it also maintains stemness in cancer stem cells.

## 5. HIF-1, the Warburg Effect, and the Innate Immune Response in COVID-19

During RNA virus lung infection such as coronavirus, viral RNAs are detected by sensors, thus inducing IFN-regulatory factor- (IRF-) 3, IRF-7, and NF-*κ*B-mediated expression of interferon- (IFN-) *α* and IFN-*β* as well as of other proinflammatory cytokines [22]. In SARS-CoV and MERS-CoV infections, the antiviral response is type-I IFN-mediated [23]. IFNs binding to their receptors lead to the phosphorylation of signal transducer and activator of transcription- (STAT-) 1 and STAT-2 transcription factors; this allows their migration to the nucleus where they bind to the promoter region of target genes, including iNOS and IL-12, associated with proinflammatory M*Φ*/MG activation [24]. In line with this, it has been demonstrated that STAT-1 is required for M1 polarization [25]. Interestingly, IFN-*α*/*β* signaling is essential for limiting virus dissemination throughout the central nervous system (CNS) thanks to the interaction between the IFN-*α*/*β* and the IFN-*γ* pathways, these being essential components of virus control in achieving optimal IFN-*γ* antiviral M*Φ*/MG responsiveness [26]. Notably, the clinical outcome of COVID-19 can be influenced by the time and the extent of the IFN response; in fact, mild and moderate SARS-CoV-2 infection has been associated with a stronger early type-I IFN response, compared to the lower IFN response observed in severe patients [27].

Type-I IFN-induced high expression of STAT-1 and STAT-2 leads to M1-like M*Φ* polarization. It has been proposed that the polarization of pulmonary M*Φ* towards the proinflammatory phenotype contributes to controlling viral replication. Indeed, in various viral respiratory diseases such as SARS and influenza, viral infection causes significant depletion of M1 M*Φ* through apoptosis and necrosis facilitating viral replication [28]. However, it is also known that, although M1 are important in fighting the virus, a balanced activation of the M2 M*Φ* subsets is essential for limiting immunopathological reactions. Liao et al. indicated that bronchoalveolar lavage fluid (BALF) from patients with severe COVID-19 infection had elevated M1 M*Φ*, while BALF from moderately infected patients and healthy controls contained a higher frequency of M2-like M*Φ* [29]. The initial impact of the viral infection and the response of the pulmonary parenchyma innate immunity is closely linked to the presence of M1 M*Φ* and also to the subsequent appearance of local M2 M*Φ*. In fact, the action of M1 subsets to counteract viral infection is necessary, even though their excessive and persistent presence can lead to severe forms of pneumonia by self-feeding the lung inflammation.

The SARS-CoV-2 virus affects the lungs and blood vessels and also the CNS where it mainly induces a chronic and pronounced inflammatory response and a cytokine storm that indirectly damages the CNS [30]. SARS-CoV-2 is neuroinvasive and may spread from the periphery to the brain, probably by the retrograde axonal transport through the vagus nerve and the olfactory nerve but also through the enteric nervous system and the hematogenous pathway. The angiotensin-converting enzyme-2 (ACE-2) receptor for SARS-CoV-2 is expressed in the capillary endothelium of the CNS; therefore, SARS-CoV-2 could bind and break the blood-brain barrier (BBB) to enter the CNS similarly to previous SARS coronaviruses. Moreover, it has been found that ACE-2 receptors are also expressed on various neuronal types, on astrocytes, and on microglia.

Although the pathological basis of the neurological damage in COVID-19 is still poorly understood, it seems that the neurological symptoms of COVID-19 infection are due to the massive systemic immune response and the subsequent proinflammatory cytokines and cytotoxic T lymphocyte infiltration into the CNS through the BBB, as well as to the strong activation of the resident immune cells, i.e., MG and astrocytes. In particular, it has been proposed that cytokines produced by MG contribute to disrupting the homeostasis of the CNS more than systemic inflammatory molecules do. Notably, if MG is in a “primed status,” e.g., by conditions which contribute to systemic inflammation such as diabetes, ischemic conditions, and arthritis (all particularly frequent in the elderly), then a secondary stimulus such as a viral infection might further activate “primed” MG. This might explain why the elderly have a higher risk of experiencing neurological and cognitive disabilities in COVID-19 [31, 32]. As stated, the excessive proinflammatory MG activation seems to be the main cause of neuropathological damage in COVID-19 patients. Indeed, although SARS-CoV-2 could be detected in the brains of most examined patients where it might, in principle, have direct cytopathic effects disrupting the complex neural circuits, such effects are rare [33]. Moreover, the activation of the glycolytic pathway in chronic activated M1 MG might cause acidosis in the brain which can contribute to the neuropathological manifestations of COVID-19.

Neuropathological manifestations in COVID-19 are long-term secondary or bystander pathologies developing much later than the primary disease. They include anosmia, hypogeusia, headache, nausea and altered consciousness, seizures, stroke and acute cerebrovascular accidents, encephalitis and demyelinating disease, and, possibly, loss of control of respiration exacerbating hypoxemia. The lack of O_2_ caused by damaged lung epithelial cells may cause—in critical COVID-19 patients—hypoxia disorders in the entire body including the CNS and subsequent cerebral damage.

As above stated, the M1 M*Φ* polarization is strictly dependent on the metabolic shift to glycolysis and to HIF-1*α* signaling. Therefore, although specific studies are necessary and the details of M*Φ*/MG polarization kinetics during the various stages of COVID-19 and its association with pathogenesis need to be unraveled, it is highly probable that M*Φ*/MG involved in the first phases of SARS-CoV-2 infection are M1-polarized and HIF-1*α* expressing and perform aerobic glycolysis. Notably, underlining the connection between viral infection, IFN production, STAT-1/2 activation, and M1 polarization, it has been found that sixteen enzymes involved in the glycolytic pathway are upregulated by STAT-1, which is typically induced by the virus [34]. Moreover, the increased glycolysis in a single COVID-19 patient has been explored by using 18F-fluorodeoxyglucose positron emission tomography (FDG PET) which identified FDG accumulation in the right paratracheal, right hilar lymph nodes, and bone marrow [35]. Ayres provides an interesting speculative dissertation on the relationship between metabolism and COVID-19 and on the potential relevance of glycolysis, using as his basis a parallelism between the pathophysiology of some metabolic abnormalities and the disease course of COVID-19 [36].

## 6. Hypoxia-Dependent Mechanisms in Cancer and Speculation on COVID-19 Pathogenesis

Besides the induction of the M1 proinflammatory response triggered by the virus through IFNs and STAT-1/2 and leading to normoxic HIF-1 activation and to aerobic glycolysis, COVID-19 infection also induces severe hypoxia conditions. Hypoxia, in turn, is the classical inducer of HIF-1 with subsequent inflammatory cytokine production and glycolysis enhancement. Therefore, the COVID-19 hypoxic conditions and the following HIF-1-dependent gene expression likely potentiate and exacerbate M1 polarization and the degree of inflammation of ACE-2-positive tissues [37], thus possibly reducing or delaying the shift to M2 M*Φ* subsets necessary for tissue repair. Hypoxia might therefore be considered pathogenic for COVID-19 and also for the complications in noble tissues sensitive to the degree of tissue oxygenation, such as in the brain, found in severe forms of COVID-19 [38].

In COVID-19, besides the alveolar damage, viral attack involves the endothelium and causes coagulation. Autopsy data have confirmed that the lung parenchyma injury is characterized by alveolar wall thickening, vascular hyperpermeability, and inflammatory cell infiltration [39]. Massive pulmonary embolism and deep thrombosis in the prostatic venous plexus have also been described due to fibrin thrombi associated with high levels of D-dimers in the blood configuring the disseminated intravascular coagulation (DIC) [39]. The microthrombi were prevalently identified in the areas of diffuse alveolar disruption and were associated with diffuse endothelial damage, thus helping to explain the severe hypoxemia characterizing the acute respiratory distress syndrome (ARDS) in COVID-19 patients [4, 19]. The hypoxemia favors tissue hypoxia at the sites of infection where the amount of O_2_ available for each cell is reduced [37].

Although the transcriptionally regulated tissue adaptation to hypoxia in the pathophysiology of SARS-CoV-2 infection requires fuller investigation, our knowledge of the hypoxia-mediated immunoescape mechanisms occurring in tumor cells corroborates speculation concerning the potential role of hypoxia-mediated mechanisms in causing inadequate immune response against SARS-CoV-2 at the infection site. The hypoxic microenvironment is a pathophysiologic condition generated during SARS-CoV-2 infection which recalls that occurring in cancer disease. Hypoxia arises in cancer tissue through the uncontrolled and rapid proliferation of cancer cells; the parallel lack of sufficient vascularization leads cancer cells to rapidly consume O_2_ and nutrients and to create a hypoxic microenvironment [40]. Similarly, hypoxia arises in tissues infected by SARS-CoV-2 through the diffusion of a rapid and uncontrolled inflammation and through a parallel lack of O_2_ caused by the thrombotic event and by the alveolar damage, all inducing a hypoxic microenvironment. Interestingly, hypoxia stabilizes HIF-1*α* and promotes the glycolytic phenotype in cancer cells whereas, in the surrounding nontumor tissue, the prolonged lack of O_2_ inhibits regular cell function [7]. Moreover, it has recently been found that hypoxia-induced HIF-1 enhances the overexpression of programmed death ligand-1 (PDL-1) on the tumor cell surface. PDL-1 binds to programmed cell death protein 1 (PD-1) which is expressed by T-cells, thus preventing the cytotoxic activation of tumor-infiltrating T-cells and promoting tumor cell survival thanks to immune system surveillance escape [5, 41]. Recently, it has been suggested that similar mechanisms of hypoxia-dependent immune system escape based on the PD pathway might also occur in COVID-19 [42–44]. Further studies in this regard—e.g., aimed at evaluating PDL-1/PD-1 modulation in COVID-19 patients—might be desirable in order to clarify this important issue and to gain insight into why in COVID-19 patients with an altered immune response the occurrence of hypoxia and inflammation (also present in other diseases such as severe influenza) leads to an undesirable outcome.

In this context, it is interesting to note that some researchers have observed that cancer patients undergoing treatment with inhibitors of the PD pathway—immune checkpoint inhibitors (ICI) used to treat solid tumors such as melanoma, lung cancer, renal carcinoma, urothelial cancers, and head and neck carcinoma—could be more immunocompetent than cancer patients undergoing chemotherapy [41]. Notably, the evaluation of the potential therapeutic value of ICI recently tested for cancer treatment in restoring cellular immunocompetence in COVID-19 is attracting interest; indeed, a trial with the ICI anti-PD1 camrelizumab is ongoing in SARS-CoV-2-infected patients [45].

Here, we wish to emphasize these similarities with other known conditions of hypoxia since they might provide useful insight during the disease's late aggressive stages (characterized by high hypoxia and inflammation and by a high viral burden overcoming the patient immune response) and when alternative routes aimed at counteracting the pO_2_ lowering are required.

Notably, comparative evaluation between COVID-19 and non-COVID-19 pneumonia patients suggested an impact of the SARS-CoV-2 infection on lymphocyte subset count which decreases in COVID-19. In particular, in SARS-CoV-2 patients, the B lymphocyte subset exhibits the most significant decrease compared to in non-SARS-CoV-2 pneumonia-infected patients and may fail to restrict the virus expansion [46]. The reduced levels of B lymphocytes in SARS-CoV-2 patients might be associated with HIF-1 activation [47]. In fact, hypoxia alters B cell physiology and function leading to reduced proliferation and increased B cell death. Moreover, hypoxia and the constitutive HIF-1 activation impair the generation of high-affinity IgG antibodies [48], and HIF-1*α* modulates recombinant Ig isotype variation in B cells, thus influencing memory recall. Therefore, the dysfunctional antibody production occurring in COVID-19 might possibly be related to B cell hypoxic damage [49]; a jammed antibody production by B cells caused by hypoxia and prolonged HIF-1 signaling activation could explain why some subjects, despite having contracted the virus, do not have a neutralization immunoconversion [50].

## 7. Potential Therapies for COVID-19 Targeting Hypoxia-Related Pathways

### 7.1. Targeting HIF-1 and/or Switching the Metabolism

Based on these premises, acting on aerobic glycolysis and/or on HIF-1 as possible therapeutic targets would have the effect of containing the presence and the activity of proinflammatory M1, in the right context, while favoring M2 polarization, thus inducing the resolution of the inflammatory process and promoting tissue repair.

The concept of M*Φ*/MG reprogramming to promote anti-inflammatory/regenerative M2 polarization able to reduce inflammation might offer a new therapeutic approach to both promote tissue healing and reduce inflammation. Manipulating immune cell metabolism in order to regulate the immune cell development can enhance or temper the immune response and drive M*Φ*/MG polarization and function, which might be useful for the potential treatment of several diseases, including COVID-19 [16]. As reviewed by De Santa et al., calorie restriction stimulates adaptive metabolic changes with many positive effects such as lifespan extension and delayed age-associated disease onset. It has been found that calorie restriction in mice leads to M2 polarization. Moreover, nutrients impinging on metabolism such as resveratrol, vitamin D, pomegranate, and its polyphenols, grape seed-derived polyphenols (proanthocyanidolic oligomers), inhibit M1 activation and promote M2 activation. Also, some sirtuins seem to be able to modulate metabolism promoting an M1-to-M2 transition.

We also speculate that a possible pharmacological method for supporting the mitochondrial respiration could be the strong inhibition of the *β*-adrenergic receptors (*β*-ARs)/uncoupling protein 1 (UCP1) axis in the tissues where it is activated. *β*-ARs control thermogenesis by increasing the expression of UCP1 which is a member of the mitochondrial anion carrier family located on the internal mitochondrial membrane and predominantly expressed in brown adipocyte tissue [51]. *β*3-AR is the main trigger of UCP1 which uncouples the activity of the respiratory chain from the synthesis of ATP, thus releasing energy as heat for an adaptive thermogenesis [52]. The selective *β*3-AR antagonism, by using SR59230A, reduces heat production favoring the synthesis of ATP. Similar effects were observed with the use of genipin specifically inhibiting UCP2—ubiquitously expressed in tumor cells—which has been found to reduce the glycolytic pathway activation in cancer cells, shifting their metabolism toward the mitochondrial pathway [53].

### 7.2. Targeting Hypoxia

The pathogenesis framework here described suggests that one of the pathophysiologic mechanisms in SARS-CoV-2 disease could be represented by the grade of hypoxia. The consolidation of a hypoxic microenvironment could be antagonized by opportune pharmacological actions aimed at improving O_2_ supply (i) indirectly—by reducing the exuberance of the innate response and limiting the release of cytokines in the right time frame—and (ii) directly. Currently, no country has licensed any specific pharmaceutical treatments for COVID-19, in particular for the late aggressive stages of this disease characterized by high hypoxia and inflammation.

To limit hypoxia-inducible complications of SARS-CoV-2 pneumonia, the pO_2_ in the plasma must increase [54]. To this end, O_2_ supplementation via nasal cannulation/mechanical ventilation is performed. Although not used for pneumonia, an additional modality to increase the pO_2_ in the plasma is the hyperbaric O_2_ therapy (HBOT). By HBOT, patients are treated with 100% O_2_ at pressures greater than atmospheric pressure, this increasing the amount of O_2_ dissolved in the plasma, thereby improving its delivery to stressed tissues reached by the blood flow [54]. Here, we discuss about another way to increase O_2_ delivery to tissues which might be the administration of ozone (O_3_) (*ozone therapy*) [55, 56]. O_3_ has a short half-life and must be produced at the time of use by equipment which transforms medical O_2_ into O_3_. One of the most important routes of O_3_ administration is direct intravenous by different delivery methods [55, 57, 58]. For example, a commonly accepted delivery method is the ozonated autohemotherapy by which a precisely controlled O_2_/O_3_ gaseous mixture is injected into the same volume of blood drawn from a patient and allowed to mix with it. The ozonized blood is then intravenously infused back into the same patient [59–61].

The main action of the *ozone therapy*—reported to be exceptionally safe—is the O_3_ germicidal ability to reduce the infectivity of a wide range of pathogens including viruses, by lipid peroxidation, viral capsid damage, and inhibition of virus replication [57, 62]. Atoxic doses of O_3_ have also been found to stimulate the innate immune system besides being a strong anti-inflammatory and antioxidant molecule counteracting the oxidative stress by upregulating the expression of antioxidant enzymes (glutathione peroxidase, catalase, and superoxide dismutase) [63–65]. Thus, many authors have recently proposed that the *ozone therapy* might be cytoprotective and improve clinical conditions caused by SARS-CoV-2 [60, 63, 66–68]. Moreover, besides these effects, various data describe the role of ozonated autohemotherapy in treating hypoxia by correcting the hypoxemia and improving O_2_ delivery and tissue oxygenation [55, 58, 59, 68]. In fact, once in the blood, the O_3_ reacts with organic compounds containing double bonds (i.e., polyunsaturated fatty acids) and generates messengers such as aldehydes derived from the unsaturated fatty acid peroxidation and hydrogen peroxide. The hydrogen peroxide increases the glycolysis rate into erythrocytes and enhances the production of 2,3 di-phosphoglycerate deriving from 1,3-diphosphoglycerate obtained by glycolysis, thanks to the enzyme diphosphoglycerate mutase [68]. 2,3 di-phosphoglycerate, in turn, is able to reduce the affinity of the hemoglobin for the O_2_, thus increasing the amount of O_2_ released from hemoglobin to the tissues [65, 69, 70].

The reduction of the degree of hypoxemia is an important goal in the treatment of severe symptomatic SARS-CoV-2 patients; this review highlights the need to treat hypoxia. On the other hand, O_2_ supply, if not properly calibrated, can determine side effects linked to an increased tissue oxidative damage by local release of ROS. Not only is the increment of ROS one of the immune system's strategies for fighting infections typically during the proinflammatory M1 phase but also it presents a serious risk of tissue damage, especially in noble parenchyma. In this context, it is necessary to concentrate on those patients whose oxidative system has previously collapsed through comorbidity or by aging. Accordingly, before O_2_ supply, it would be useful to verify the health of the oxidative system in real time through a blood sample [71, 72]. For example, the accumulation of methylglyoxal adducts indicates both a prevalent glycolytic metabolism and glutathione defensive system failure [72]. Through an oxidative screening of the blood, it would be possible to select the SARS-CoV-2 patients with a preserved antioxidant defense potentially benefitting from a therapy aimed at counteracting the oxidative mitochondrial phosphorylation shut down by hypoxia and inflammation. An oxidative screening on a blood sample will give information regarding all body tissues, whereas a specific analysis of the oxidative stress at each tissue level, although desirable, would require a tissue biopsy and would be invasive. In the future, the concomitant evaluation of tissue-specific markers in a blood sample might provide insights into a specific tissue. For example, the endothelial damage is typical of this disease; circulating endothelial cells (CEC) have been found in the blood of COVID-19 patients [73]. Therefore, the evaluation of the oxidative stress on CEC might be a way to evaluate the oxidative stress on specific cells by using a liquid biopsy. The analysis of specific exosomes, whose tissue origin could be identified by peculiar markers, would potentially shed light on that specific tissue's oxidative stress.

## 8. Concluding Remarks

Manifestations displayed by COVID-19 patients led us to consider the central role of HIF-1 and metabolic reprogramming in the inflammatory response typical of this disease, characterized by lung dysfunction. The side effects of the hypoxia/HIF-1 signaling triggered during the SARS-CoV-2 infection are a potentially important area of research for pharmacological applications. Hopefully, further studies will disclose M*Φ*/MG heterogeneity in COVID-19 and the metabolic pathways associated with M*Φ*/MG polarization, thus identifying novel immunometabolic molecular targets and contributing to therapeutic interventions. Cancer disease might provide insights into COVID-19 pathophysiology; however, defining the precise role of hypoxia in the development of the COVID-19 syndrome severity still requires further study. Nevertheless, we cannot avoid the conclusion that hypoxemia and hypoxia are sides of the same coin and that by trying to solve one, inevitably, we also affect the other.

## Abbreviations

ACE-2: Angiotensin-converting enzyme 2
ARDS: Acute respiratory distress syndrome
BBB: Blood-brain barrier
*β*-ARs: *β*-Adrenergic receptors
CARKL: Carbohydrate kinase-like
CEC: Circulating endothelial cells
CNS: Central nervous system
COVID-19: Coronavirus disease 2019
DIC: Disseminated intravascular coagulation
FDG PET: 18F-fluorodeoxyglucose positron emission tomography
GLUT1: Glucose transporter type 1
HBOT: Hyperbaric O_2_ therapy
HIF-1: Hypoxia-inducible factor-1
ICI: Immune checkpoint inhibitors
IRF: IFN-regulatory factor
Idh1: Isocitrate dehydrogenase 1
iNOS: Inducible NO synthase
IFN: Interferon
IL: Interleukin
Irg1: Immunoresponsive gene 1
M*Φ*: Macrophage
MG: Microglia
NF-*κ*B: Nuclear factor kB
NO: Nitric oxide
NOX: NADPH oxidases
pO_2_: Oxygen partial pressure
OxPhos: Oxidative phosphorylation
PDK1: Pyruvate dehydrogenase kinase-1
PFKFB3: Phosphofructo-2-kinase/fructose-2,6-biphosphatase 3
PHDs: Prolyl hydroxylases
PKM2: Pyruvate kinase M2
PPP: Pentose phosphate pathway
PD-1: Programmed cell death protein 1
PDL-1: Programmed death ligand-1
pVHL: von Hippel-Lindau tumor suppressor
ROS: Reactive O_2_ species
SARS-CoV-2: Severe acute respiratory syndrome coronavirus
TNF: Tumor necrosis factor
STAT: Signal transducer and activator of transcription
TCA: Tricarboxylic acid cycle
UCP1: Uncoupling protein 1
VEGF: Vascular endothelial growth factor.
